# 肺腺癌患者接受免疫检查点抑制剂治疗后引发巨细胞病毒胃炎1例病例报道

**DOI:** 10.3779/j.issn.1009-3419.2025.102.31

**Published:** 2025-08-20

**Authors:** Xiaoyan SI, Bei TAN, Xin CHENG, Mengzhao WANG, Xiaotong ZHANG, Li ZHANG

**Affiliations:** ^1^100730 北京，中国医学科学院，北京协和医学院，北京协和医院呼吸与危重症医学科（斯晓燕，王孟昭，张晓彤、张力）; ^1^Department of Pulmonary and Critical Care Medicine; ^2^消化科（谭蓓）; ^2^Department of Gastroenterology; ^3^核医学科（程欣）; ^3^Department of Nuclear Medicine, Peking Union Medical College Hospital, Chinese Academy of Medical Sciences and Peking Union Medical College, Beijing 100730, China

**Keywords:** 肺肿瘤, 免疫检查点抑制剂, 巨细胞病毒胃炎, 病例报道, Lung neoplasms, Immune checkpoint inhibitors, Cytomegalovirus gastritis, Case report

## Abstract

免疫检查点抑制剂（immune checkpoint inhibitors, ICIs）已广泛应用于实体肿瘤和血液系统肿瘤的治疗。ICIs引起的不良反应逐渐被重视。ICIs治疗后出现巨细胞病毒胃炎较为少见。本文结合文献复习报告1例晚期肺腺癌患者在使用帕博利珠单抗（Pembrolizumab）后出现反复上腹痛、呕吐，通过胃镜活检确诊为巨细胞病毒胃炎，患者接受抗病毒治疗后症状好转。在ICIs治疗过程中，需注意上腹痛症状的鉴别诊断，警惕巨细胞病毒胃炎。巨细胞病毒胃炎和免疫相关胃炎难以通过症状来鉴别，胃镜活检非常重要。

## Abstract (en)

Immune checkpoint inhibitors (ICIs) have been approved for the treatment of a variety of solid tumors and hematological malignancies. Adverse reactions caused by ICIs have been gradually focused on. Cytomegalovirus (CMV) gastritis after ICIs treatment is relatively rare. Here we reported a case of advanced lung adenocarcinoma who experienced recurrent upper abdominal pain and vomiting after Pembrolizumab treatment. CMV gastritis was diagnosed through gastroscopy. The patient's symptoms improved after antiviral treatment. During the treatment of ICIs, attention should be paid to the differential diagnosis of upper abdominal pain symptoms, and vigilance should be maintained against CMV gastritis. It is difficult to differentiate CMV gastritis and immune-related gastritis judging from symptoms, and gastroscopy is important for differential diagnosis.

免疫检查点抑制剂（immune checkpoint inhibitors, ICIs）已成为治疗恶性肿瘤的重要药物。免疫相关的胃肠道不良反应是常见的免疫治疗相关副作用，其中以结肠炎最为常见。但整个胃肠道，包括小肠、胃、食管也可能会受累。此外，还有文献^[1-3]^报道了ICIs治疗过程中慢性或潜伏性感染的再激活。本研究报道在北京协和医院诊治的1例使用ICIs后出现巨细胞病毒（cytomegalovirus, CMV）胃炎的晚期肺腺癌病例，以引起临床关注。

## 1 病例资料

患者女，57岁，2019年12月诊断为右肺腺癌，IV期，Kirsten大鼠肉瘤病毒癌基因同源物（Kirsten rat sarcoma viral oncogene homolog, KRAS）G12A突变，程序性细胞死亡配体1（programmed cell death ligand 1, PD-L1）肿瘤细胞阳性比例分数为80%。2019年12月起予6个周期紫杉醇（Paclitaxel）、卡铂（Carboplatin）、帕博利珠单抗（Pembrolizumab）治疗。评估疗效为部分缓解。2020年5月至2021年2月予11个周期帕博利珠单抗维持治疗。2021年2月起，患者出现反复的上腹部疼痛，伴恶心、呕吐胃内容物，无呕血，无便血、黑便。血常规：白细胞5.86×10^9^/L，中性粒细胞4.3×10^9^/L，淋巴细胞0.8×10^9^/L，血红蛋白137 g/L。便潜血试验阳性。血清抗CMV IgM抗体阴性，抗CMV IgG抗体阳性。血CMV DNA阴性。正电子发射计算机断层显像（positron emission tomography/computed tomography, PET/CT）显示整个胃壁明显增厚，弥漫代谢不均匀摄取增加，标准化摄取值最大值为9.9。胃镜显示全胃黏膜出血糜烂，表面覆盖白苔。胃壁活检病理显示胃黏膜重度慢性活动性炎症，个别隐窝萎缩，偶见腺腔内脓肿，可见CMV包涵体，幽门螺杆菌检测结果为阴性。原位杂交显示CMV阳性。临床诊断为CMV胃炎，予更昔洛韦（Ganciclovir）250 mg bid治疗3周，患者腹痛和恶心呕吐症状逐步减轻。3个月后复查胃镜显示黏膜病变明显好转，原位杂交显示CMV阴性（图1）。

**图1:**
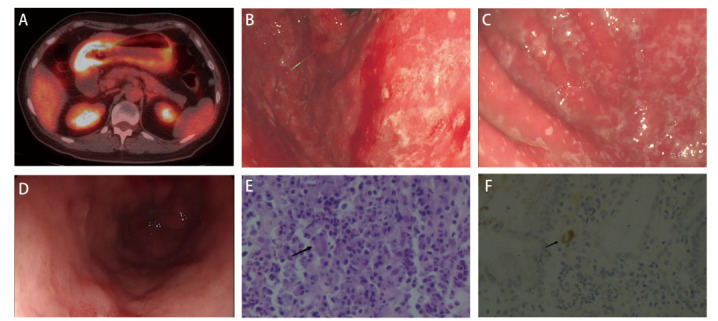
影像学及病理图片。 A：PET/CT显示整个胃壁明显增厚，弥漫代谢不均匀摄取增加；B、C：胃镜显示全胃黏膜出血糜烂，表面覆盖白苔；D：抗病毒治疗后复查胃镜显示黏膜病变明显好转；E：胃黏膜活检可见细胞病毒包涵体（HE, ×400）；F：原位杂交显示巨细胞病毒阳性（×400）。

## 2 讨论

CMV是一种常见的疱疹病毒。在急性感染消退后，CMV可形成潜伏性感染。CMV胃炎在免疫正常和免疫抑制的人群中都可以出现，主要表现为消化道出血、上腹痛和发热^[4]^。胃镜表现为溃疡或糜烂，病理上可以看到CMV包涵体。治疗首选更昔洛韦，疗程通常为3至6周，酌情可延长为8至16周^[5,6]^。

在处理免疫相关不良反应使用激素和免疫抑制剂治疗后，因为患者处于免疫抑制状态，可能会出现CMV感染。此外，也有病例报告^[6-8]^提示帕博利珠单抗治疗后患者在没有免疫抑制的状态下出现CMV胃炎，推测原因可能是ICIs治疗后潜伏的CMV再激活。其中1例患者为CMV胃炎合并幽门螺杆菌感染^[6]^。本病例在使用帕博利珠单抗单药治疗中出现CMV胃炎，并没有使用免疫抑制药物，而且CMV IgG阳性，考虑可能与CMV再激活相关。本病例未合并幽门螺杆菌感染。研究^[9]^表明，ICIs可以增强病毒特异性T细胞活性。ICIs引起病毒特异性T细胞的恢复和对CMV抗原的过度炎症反应，导致免疫重建炎症样综合征^[10]^。本病例与之前报道的病例^[6]^外周血CMV DNA均为阴性，再次表明血CMV DNA阴性不能排除组织CMV疾病，组织活检则是诊断金标准。

ICIs治疗后的免疫相关胃炎也有报道^[11-15]^。免疫相关胃炎的病理提示固有层淋巴细胞浸润和上皮内CD8^+^ T淋巴细胞增加。此外还有免疫相关胃炎和CMV胃炎共存的病例报道^[16]^。在这项病例报道中，胃镜活检病理提示固有层单核炎性细胞浸润，隐窝凋亡，但患者单用抗病毒治疗后症状即缓解，并没有使用糖皮质激素，故诊断免疫相关胃炎尚不确定。

ICIs治疗后免疫相关胃炎与CMV胃炎因为症状相似，鉴别主要依靠胃镜活检病理。CMV胃炎病理可见CMV包涵体，原位杂交显示CMV阳性。治疗上，糖皮质激素治疗免疫相关胃炎有效，而CMV胃炎需要抗病毒治疗。

综上，在ICIs治疗过程中，需注意上腹痛症状的鉴别诊断，警惕CMV胃炎。CMV胃炎和免疫相关胃炎难以通过症状来鉴别，建议行胃镜活检。
